# Correction to ‘Limited thermal plasticity and geographical divergence in the ovipositor of *Drosophila suzukii*’

**DOI:** 10.1098/rsos.200241

**Published:** 2020-03-04

**Authors:** Ceferino Varón-González, Antoine Fraimout, Arnaud Delapré, Vincent Debat, Raphaël Cornette

*R. Soc. open sci.* **7**, 191577. (Published Online 22 January 2020) (doi:10.1098/rsos.191577)

This correction refers to an error in the caption for figure 6. The copyright information was missing; this has now been corrected.

